# The “One Piece” Autologous Tuberosity Graft: A Contemporary Concept in Ridge Preservation

**DOI:** 10.1155/2020/3945076

**Published:** 2020-02-13

**Authors:** Ronald Younes, Carla Maria Khairallah

**Affiliations:** ^1^Department of Oral Surgery, Faculty of Dental Medicine, Saint Joseph University of Beirut, Beirut, Lebanon; ^2^Craniofacial Research Laboratory-Division of Oral Biology, Saint Joseph University of Beirut, Faculty of Dental Medicine, Beirut, Lebanon

## Abstract

Tooth extraction causes major changes to the ridge, rendering implant placement a more challenging procedure. Proper management of the socket is necessary to ensure sufficient bone and soft tissue for a successful implant-supported prosthesis. This article presents a practical approach for autologous hard and soft tissue grafting. A soft and hard tissue graft is harvested in one piece from the maxillary tuberosity and stabilized in the extraction socket.

## 1. Background

Placing an implant in the aesthetic zone is a challenging procedure. Following a tooth extraction, dimensional changes to the ridge occur, as a result of the healing process. In fact, based on a systematic review in 2012, 29 to 63% of horizontal bone loss and 11 to 22% of vertical bone loss, corresponding to 3.79 ± 0.23 mm and 1.24 ± 0.11 mm, respectively, occur 6 months after an extraction [1]. And at 12 months, as much as 50% reduction in width of the alveolar ridge occurs [2].

Several treatments following tooth extraction are available: immediate or delayed implant placement with or without bone and/or soft tissue augmentation, and socket preservation followed by a delayed implant placement.

In order to facilitate the choice of treatment, a new classification in 2016 divided single-rooted tooth extraction sockets into three grades, depending on the buccal plate morphology, the apical bone topography, and the interproximal bone available. Thus, a treatment is chosen according to the socket's classification. Therefore, grades I and II with thick biotype are eligible for immediate implant placement, while grade II with thin biotype and grade III, a socket preservation is advisable [3].

While a number of publications showed a significantly lower survival rate of immediately placed implants compared to delayed implants inserted into healed sockets [4–7], socket preservation techniques have been used frequently to idealize the delayed implant placement in sufficient amounts of bone, with an ideal implant position, providing a proper emergence profile for an esthetic and functional prosthesis. Several biomaterials have been applied in alveolar ridge preservation, alone or in combination. They included xenografts [8, 9], allografts [10, 11], alloplastic material, synthetic magnesium-enriched hydroxyapatite [12], recombinant morphogenetic protein-2 (rh-BMP2) [13], autologous bone marrow [14], autologous bone particles [15], and autologous blood-derived products (PRF, PRP) [16, 17]. Those grafting materials were used as socket fillers with or without collagen membrane [8, 10], and/or free gingival grafts [18] as socket sealers.

In general, bone substitutes show complete osseous integration, but they seem to decelerate bone regeneration in the early healing phases and reduce the proportion of vital bone when compared to autologous grafts [19, 20]. Xenografts are known to require long periods of healing and resorption, leading usually to high numbers of residual graft particles at early reentries [21–23]. To the contrary, alloplastic material usually has the highest amount of vital bone and the smaller amount of residual graft material [19]. But, xenografts and allografts show more favorable results when compared to alloplastic materials, in preserving socket dimensions [19, 24]. And when attempts were made to use autologous bone chips in socket preservation, they failed to promote healing or stimulate hard tissue formation in the socket [15].

Unfortunately, to this date, no clear consensus was reached on the ideal biomaterial to be used in socket preservation.

The purpose of this article is to present a socket preservation approach using a “one-piece” autologous bone and soft tissue graft harvested from the maxillary tuberosity.

## 2. Rationale for “One-Piece” Ridge Preservation Technique

This technique is limited to sockets of single-rooted teeth.

### 2.1. Medications

- Amoxicillin with clavulanic acid (Augmentin) 1 g BID for 7 days, starting the morning of the extraction day
- Ibuprofen (Brufen) 400 TID in case of pain
- Chlorhexidine mouthwash 0.12% TID for 21 days (starting the day after the extraction)

### 2.2. Surgical Technique

*Step 1:* the patient is asked to rinse for 1 minute a solution of 0.2% chlorhexidine. Afterwards, local anesthesia is administrated. Atraumatic extraction of the tooth, preserving the alveolar bone and the surrounding soft tissue, is performed, followed by a thorough curettage of the socket in order to remove any infected tissue. Deepithelialization of the inner part of the free gingiva surrounding the socket is made using a 15C blade or a diamond bur.

**Figure 1:**
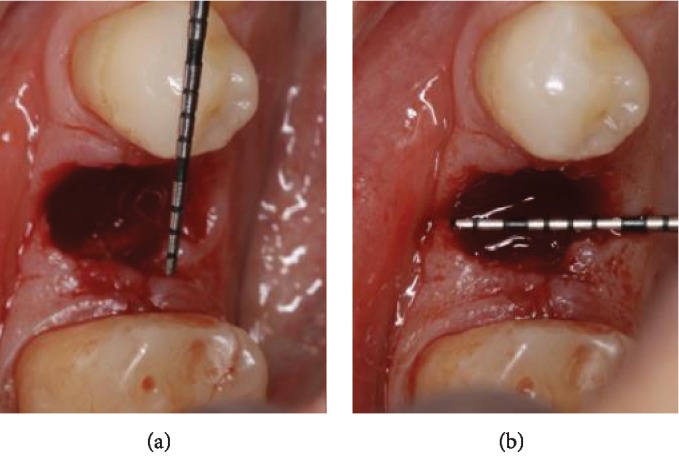
Measurements of the socket with a periodontal probe: (a) mesio-distal; (b) bucco-lingual.

*Step 2:* a periodontal probe is used to measure the dimensions of the socket in order to determine the size of the tissue punch and trephine bur to be used (Figure 1).

**Figure 2:**
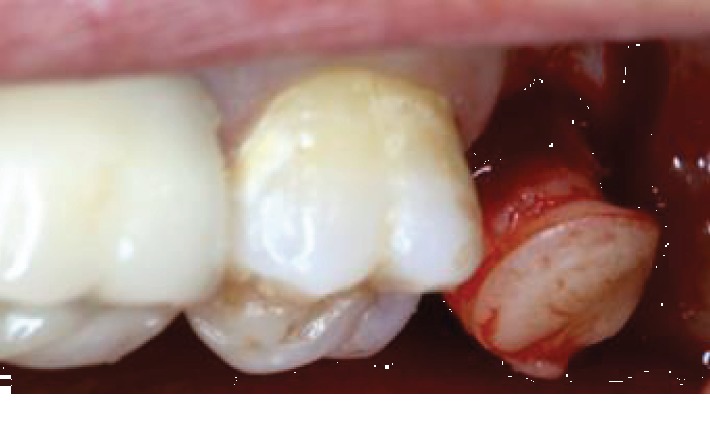
The “one-piece” tuberosity graft harvest.

A tuberosity block together with a greater palatine nerve block is performed. Using a soft tissue punch, the gingiva is demarcated. Then, the bone is perforated with a smaller trephine bur, to finally remove the gingivoosseous graft in one piece (Figure 2).

**Figure 3:**
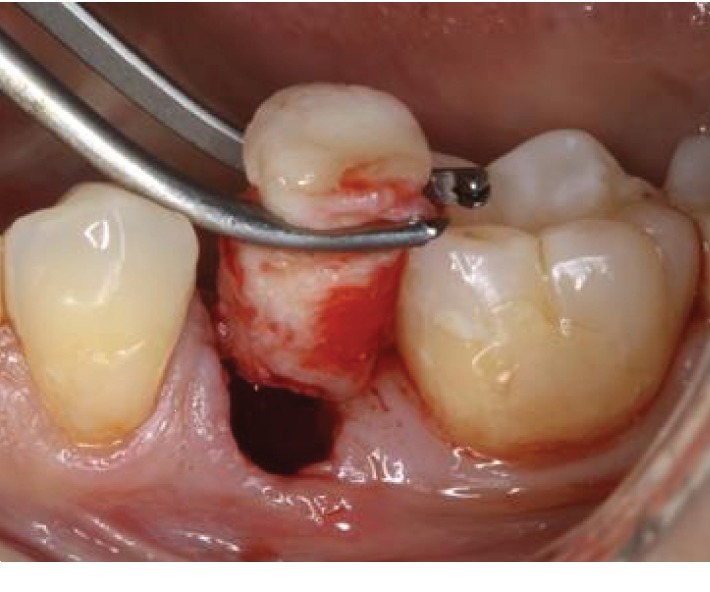
Fitting of the graft.

*Step 3:* the graft should fit adequately in the extraction socket without being compressive to the socket walls or too loose (Figure 3). Occasionally, shaping of the graft is needed, using a rongeur. The soft tissue can also be trimmed, thus avoiding overfilling of the socket.

**Figure 4:**
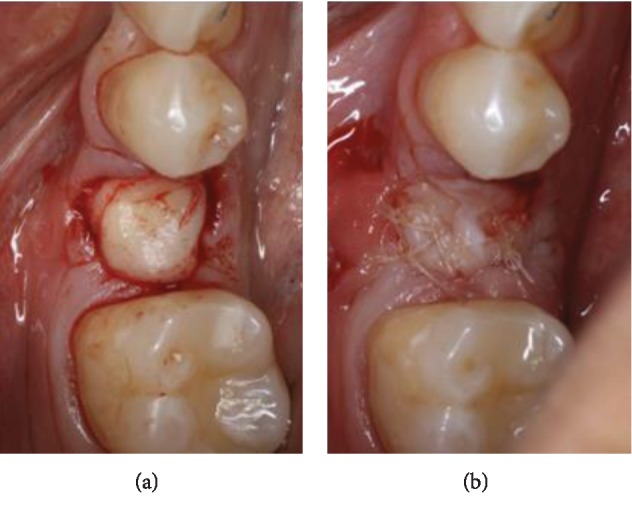
(a) Placement of the graft in the socket; (b) graft stabilized with 6/0 absorbable sutures.

*Step 4:* the composite graft is stabilized in the fresh extraction socket with simple interrupted sutures using 6/0 absorbable sutures (Figure 4).

**Figure 5:**
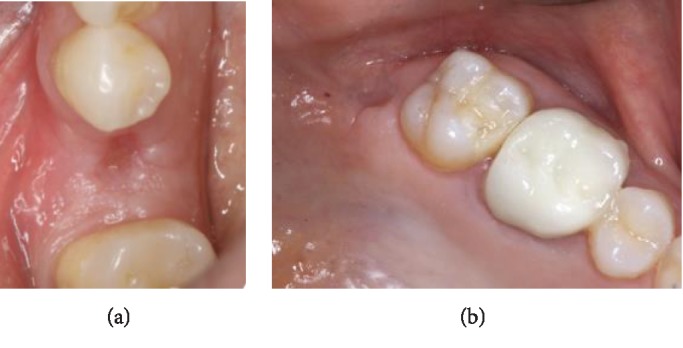
Follow-up 3 weeks post-op: (a) grafted site; (b) donor site.

*Step 5*: maintaining good oral hygiene and avoiding any removable prosthesis over the treated site (Figure 5). Duration of the healing period is 4 months, correlating with autologous bone regeneration principles.

**Figure 6:**
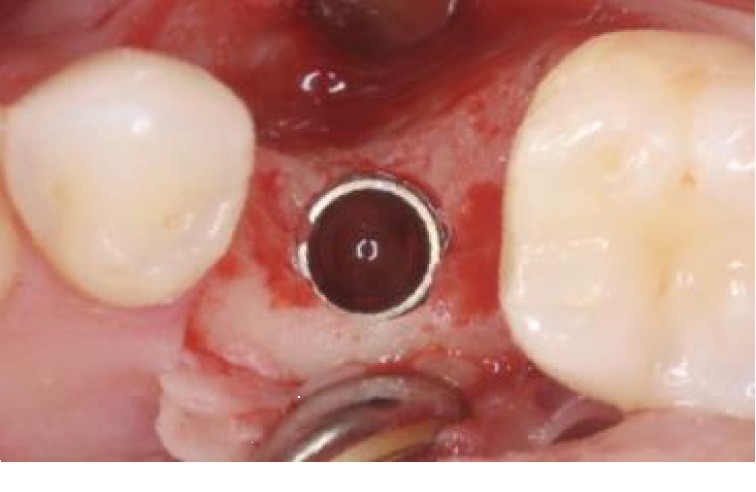
Implant placement after 4 months.

**Figure 7:**
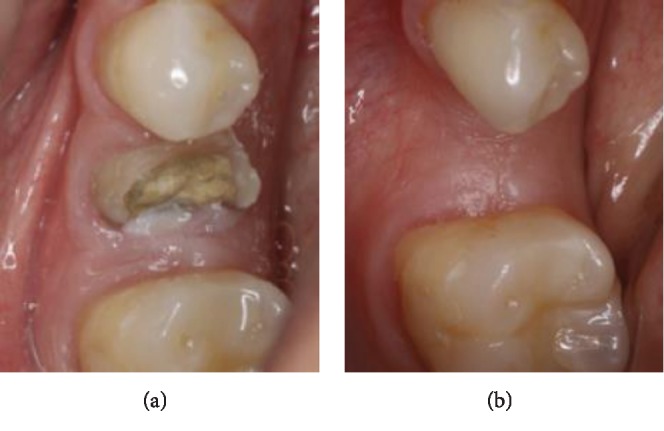
Occlusal view of the lower left second premolar site: (a) before the extraction; (b) 4 months post-op.

*Step 6*: implants could be placed at 4 months postextraction (Figures 6 and 7).

**Figure 8:**
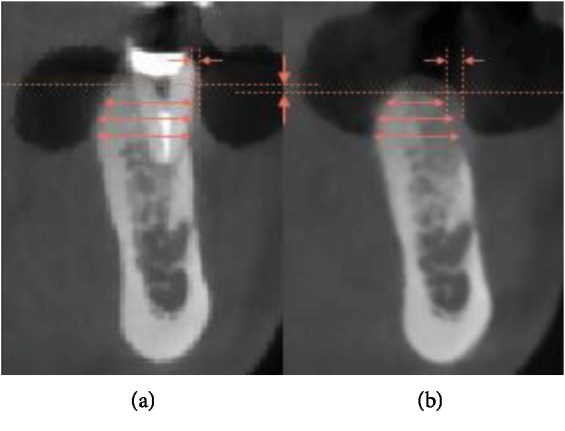
CBCT sagittal view with an illustration of radiological measurements: (a) Before the extraction; (b) 4 months post-op (vertical difference of bone levels; horizontal bone width at 2, 4, and 6 mm; soft tissue thickness at the buccal side).

A Cone-Beam Computed Tomography (CBCT) scan was taken before the extraction and 4 months after. A superimposition of both CBCTs was made to measure the socket's modifications following tooth extraction and preservation using the “one-piece” technique (Figure 8). Limited vertical and horizontal bone losses were detected. The vertical resorption was 0.71 mm. The horizontal resorption was measured at 3 different levels: 2, 4, and 6 mm from the most coronal bone peak preoperatively. The respective values were 2.71 mm, 1.29 mm, and 0.72 mm. As for the soft tissue on the buccal side, an increase of 1.23 mm in thickness was marked.

## 3. Discussion

Alveolar ridge preservation was proven to be an effective approach to decrease dimensional changes after tooth extraction [25–29].

The “one piece” technique we used satisfies the alveolar ridge preservation requirements, such as atraumatic extraction [30], flapless procedure [31], usage of bone as a filler and soft tissue as a sealer of the socket, while providing healing by primary intention.

When a mucoperiosteal flap is elevated, the blood vessels attached to the bone are severed. This will reduce the blood supply and lead to the death of local osteocytes. Hence, the surrounding mineralized tissue of the bone walls necrotize and eventually be eliminated through surface resorption [32]. This is not the case with this technique, since the graft is inserted without flap elevation, keeping the periosteum intact and the mucogingival junction (MGJ) in its original position.

Moreover, the form of the graft mimicking the root anatomy seems to prevent the ridge collapse during the healing phase. This is not the case when bone chips are used.

We have chosen to use autogenous bone and soft tissue graft. In fact, the use of autologous bone in bone regeneration is still considered the “gold standard” due to its osteogenic, osteoinductive, and osteoconductive properties [33, 34]. It has a unique advantage of retaining cell viability and containing osteoblasts and osteoprogenitor stem cells which provides true osteogenesis [34]. In this technique, the maxillary tuberosity showed no complications and better accessibility, an advantage when we know that other intraoral donor sites such as the chin and the ramus present significant postoperative bleeding, swelling, discomfort, and risk of nerve injury [35]. A limitation to its usage is the reduced available amount of bone that makes it an indication in small or medium-sized defects [36, 37]. But furthermore, the tuberosity region usually consists of a thin cortical layer and a mixture of marrow spaces, adipose tissue, and vital osteogenic cells, necessary for bone formation. The type of graft described in this case report is an inlay type of graft.

A paradigm shift in the dynamics of bone grafting has taken place in the recent decades, favoring inlay-type grafts as the one used in this case for alveolar ridge preservation techniques. Whether cortical or cancellous bone of endochondral or membranous origin, bone growth is predictable when the graft is well contained in the socket [38]. Interestingly, and fairly recently (2013), studies by Rosenthal, Buchman, and Kristoffer demonstrated that inlay-type bone grafts maintained a higher volume over the years than onlay-type bone grafts, regardless of their embryological or microarchitecture origin. Furthermore, bone grafts in the inlay position have a higher potential for revascularization, osteogenesis, osteoinduction, and osteoconduction, due to the increased bone-to-bone contact. At these sites, no resorption is noted, but on the contrary, bone growth and real expansion of the site are observed. In addition, cancellous bone results in much greater growth, regardless of its embryological origin. Afterwards, the presence of functional forces creates a favorable biomechanical matrix in which the phenotype of the grafted material is closer to that of the recipient, rather than retaining its native structural tissue. The cortical bone placed in the inlay position becomes more porous over time, while the cancellous bone becomes less and less porous.

In 1989, Whitaker demonstrated that bone grafts retain their volume significantly better in the inlay position than in the onlay position because fewer changes are caused in the overlying soft tissue. While describing the concept of biological boundaries, Whitaker emphasized the importance of the overlying soft tissue envelope. In our technique, soft tissue coverage is maintained attached to the underlying bone and stabilized in situ.

The soft tissue in the tuberosity area is formed of dense collagen fibers covered by a well-keratinized layer. This might positively affect the dimensional stability of the graft and the process of graft revascularization [39, 40]. Additionally, tuberosity grafts are known to heal fast with superior color and contour blending and a reduced pain perception by the patient [39].

To our knowledge, in 2009, Tolstunov was the first to describe the maxillary tuberosity block bone graft [36].

In 2013, da Rosa et al. have introduced a new immediate dentoalveolar restoration (IDR) technique. The IDR consists of an immediate implant placement associated with a corticocancellous block graft harvested from the tuberosity and placed in the gap between the implant and the buccal mucosa (in the absence of buccal bone wall) [41].

In 2014, the same team modified the IDR technique by adding a layer of connective tissue to the corticocancellous block, resulting in a triple graft, reserved for sockets with severe buccal bone wall damage and gingival recession. From a clinical and radiological point of view, hard and soft tissue gain had been demonstrated, leading to a satisfactory, aesthetic, and functional result [42].

Applying the “one-piece” technique for alveolar ridge preservation in this case showed a reduced hard tissue resorption and a gain of soft tissue following tooth extraction, an encouraging result when compared to previous studies reporting on dimensional changes of nontreated extraction sockets [1, 2]. This technique also contributed to a proper bone and soft tissue formation in the extraction site thus providing sufficient bone and soft tissue for a successful implant-supported prosthesis without the need of complimentary grafts.

## 4. Conclusion

The “one-piece” tuberosity graft seems to be a safe alveolar ridge preservation technique, integrating soft and hard tissue in one simple approach. It may represent a feasible, user-friendly, low-cost solution for minimizing soft and hard tissue collapse and dimensional loss following single-rooted tooth extraction.

It would be interesting to conduct future RCT studies on this technique in order to present a safe and simple approach to alveolar ridge preservation.
